# Surface Modifications of Zinc Oxide Particles with Chitosan, Polyethylene Glycol, Polyvinyl Alcohol, and Polyvinylpyrrolidone as Antibacterial Agents

**DOI:** 10.3390/polym17243283

**Published:** 2025-12-11

**Authors:** Linh Doan, Khoa Tran, Khanh G. Huynh, Tu M. D. Nguyen, Lam V. H. Tang

**Affiliations:** 1 Nanomaterials Engineering Research & Development (NERD) Laboratory, International University—Vietnam National University, Ho Chi Minh City 70000, Vietnam; 2 School of Chemical and Environmental Engineering, International University—Vietnam National University, Ho Chi Minh City 70000, Vietnam; 3 Department of Chemical Engineering, International University—Vietnam National University, Ho Chi Minh City 70000, Vietnam; 4 International University—Vietnam National University, Ho Chi Minh City 70000, Vietnam; 5 Vietnam National University, Ho Chi Minh City 70000, Vietnam; 6 School of Biomedical Engineering, International University—Vietnam National University, Ho Chi Minh City 70000, Vietnam

**Keywords:** composite, particles, polymer blend, antimicrobial, zinc

## Abstract

To investigate the effect of nanoparticle reinforcement, polymer blends (M8) comprising polyvinyl alcohol, polyvinylpyrrolidone, polyethylene glycol, and chitosan were modified using zinc oxide particles (M8/ZnO). This study introduces an M8-modified ZnO composite that offers a non-antibiotic approach relevant to antimicrobial resistance. The average particle size of the ZnO particles was determined to be 181.8 nm using scanning electron microscope (SEM) analysis. Based on the inhibition percentage, M8 has a minimum inhibition concentration (MIC) to have at least a 50% inhibition, or MIC50 value, against *Pseudomonas aeruginosa* (PA) and *Salmonella enterica* (SE) at 12.5 and 25% of M8, respectively. The MIC with at least a 90% inhibition percentage, or MIC90, of M8 against SE and PA is 25% of M8. On the other hand, the MIC50 of M8/ZnO against *SE*, *Staphylococcus aureus* (SA), and PA is 25, 12.5, and 50% of M8/ZnO, respectively. The MIC90 of M8/ZnO against SE and SA is 50% and 25% of M8/ZnO, respectively. However, M8/ZnO does not inhibit a minimum of 90% of the PA bacteria. Hence, the ratio optimization between M8 and ZnO or the usage of other particles should be considered as a topic for future study.

## 1. Introduction

Microorganisms are a persistent problem and have a negative impact on human health in hygiene products such as dental goods, domestic cleanliness, and water purification [1]. *Staphylococcus aureus* (SA), a Gram-positive bacterium, is denoted as a harmful microorganism that is present in a variety of locations, including public areas as well as medical facilities. Humans may experience several illnesses as a result of infection with *Staphylococcus aureus* in the bloodstream, skin, soft tissue, heart valves, and more [2,3]. SA is commonly found in the dry parts of the skin of atopic dermatitis patients [4,5,6,7]. Currently, antibiotics or germicides are noted as solutions that have the ability to kill SA; however, this may cause irritation to the skin or contribute to antimicrobial resistance (AMR), which has been highlighted as one of the most serious worldwide health concerns lately [7]. Other research has pointed out the ability of forming resistance to antibiotics treating *SA*, which indicates a noticeable and emergent health issue [2,8].

SA is responsible for various diseases in people, such as atopic dermatitis-related dry skin, osteomyelitis, cellulitis, etc. [1,2,3,4,5,6,7,8]. Together with SA, *Pseudomonas aeruginosa* (PA) and *Salmonella enterica* (SE) are two other common microorganisms. SE is a Gram-negative bacterium found in animal-based products, which can cause foodborne illnesses worldwide by transmitting infections from animals to humans with weakened immune systems [9,10]. Another bacterium that can affect people with severely weak immune systems is PA—a Gram-negative bacterium [11]. It is resistant to multiple antibiotic classes, which can lead to numerous treatment-related issues [12,13,14]. To eliminate these bacteria, antibiotics or germicides can be used. Although those methods are effective, they might also lead to a worldwide health concern—antimicrobial resistance (AMR) [15]. Hence, alternative techniques that do not result in AMR are being considered at this moment.

Apparently, transitional metal oxides have captured the attention of several studies due to their well-known semiconducting characteristics, which can be effectively used in various applications such as solar energy conversion, magnetic storage, and catalysts [15,16,17,18]. Moreover, the current approach of applying the antimicrobial properties of metal oxide nanoparticles is becoming more popular due to its effectiveness in various fields, such as medicine and biomedical applications [12,13,14]. It has been reported that the use of nanoparticles of a smaller size leads to greater antibacterial performance; furthermore, with this property, nanoparticles are commonly used in medical treatments along with metal oxides such as zinc, copper, or iron [9,10]. It has been reported that SA is commonly used to determine the antimicrobial properties of different nanoparticles for the prevention of human pathogens, which has been proven to be effective with ZnO particles [19,20,21]. ZnO particles (ZnOPs) are largely known as particles of an essential metal oxide, which have been used in numerous biological [22,23] as well as biomedical applications, for instance, wound dressing or antibacterial performance [24,25,26]. According to previous research, it has been proven that the size of ZnOPs greatly affects their antimicrobial characteristics; to be more specific, the smaller the size of the nanoparticles, the better their performance in preventing bacteria [27,28]. Furthermore, ZnOPs offer superior stability and a lengthy shelf life when combined with antimicrobial materials such as polymers [29,30]. ZnOPs have been shown in recent research to be effective against bacteria, which have the ability to resist many antibiotics, and have also shown promise in the prevention of biofilm formation [31,32].

Antimicrobial polymers can be created synthetically or present naturally, that is, in their use to prevent the development of microorganisms. It is believed that the functional groups, the charge on the polymers’ surface, and the molecular weight all affect the antimicrobial ability of materials [33,34,35,36,37]. Based on previous research on an optimized polymer composite, the polymers chosen for this study are polyvinyl alcohol (PVA), polyvinylpyrrolidone (PVP), polyethylene glycol (PEG), and chitosan (CS), with the concentration of polymers (PVA, PVP, and PEG) at 0.02 g/mL and CS at 0.01 g/mL, while the ratio of PVA/PVP/PEG/CS/DI is 1:1:1:1:6 (*v*/*v*/*v*/*v*/*v*) [38]. This polymer composite is called M8 in this study.

Although ZnO nanoparticles and ZnO–polymer composites have been widely studied, no work has investigated ZnO modified using this particular four-component polymer blend (PVA–PVP–PEG–chitosan), nor evaluated its antibacterial behavior using MIC assays. Because the interactions between ZnO and this unique polymer composition are unknown, producing this composite and characterizing its antibacterial profile is necessary to determine how ZnO influences M8’s performance. Therefore, this study reports the first synthesis and full characterization of the M8/ZnO composite and provides the first direct MIC comparison between M8 and M8/ZnO against SE, SA, and PA.

The importance of this work stems from the urgent global demand for non-antibiotic antibacterial materials that can reduce the spread of antimicrobial resistance.

To our knowledge, this is the first report evaluating the antibacterial efficacy of the M8/ZnO composite against *Staphylococcus aureus*, *Pseudomonas aeruginosa*, and *Salmonella enterica*, providing new comparative insights into Gram-positive and Gram-negative responses.

## 2. Experimental Methods

### 2.1. Materials

Polyvinyl alcohol (PVA), sodium hydroxide (NaOH), zinc (II) sulfate heptahydrate (ZnSO_4_·7H_2_O), and polyethylene glycol—1000 (PEG)—were purchased from Xilong Scientific Co., Ltd. (Shantou, China). Chitosan (CS) and polyvinylpyrrolidone (PVP K30) were purchased from Shanghai Zhanyun Chemical Co., Ltd. (Shanghai, China). Glacial acetic acid (AA) was purchased from RCI Labscan (Bangkok, Thailand). All materials were used as obtained.

### 2.2. Synthesis of M8

The synthesized polymer blend was synthesized based on the method of a previous publication, without any modifications [38]. This study was replicated without any modifications.

### 2.3. Synthesis of ZnO

First, 19.2 g NaOH was stirred and heated with 480 mL DI water. Then, 25.86 g of ZnSO_4_·7H_2_O was stirred with 120 mL of DI water for 15 min. Then, the salt solution was added drop-wise to the NaOH solution. The mixture was then stirred and heated continuously for 45 min. Then, the particles were washed with DI water 4–5 times. The particles were then dried in the oven at 80 °C overnight. After obtaining the dried particles, the particles were then calcinated at 750 °C at a ramp rate of 10 °C/min and held for 3 h. Finally, the dried ZnO powder was obtained.

### 2.4. Synthesis of M8/ZnO

To prepare the M8/ZnO composite, 240 mL of the previously synthesized M8 polymer blend was mixed with 0.3 g of dried ZnO powder. The mixture was first sonicated for 2 h to promote the dispersion of the ZnO particles within the polymer matrix. After sonication, the suspension was stirred for an additional 30 min to ensure homogeneity. The resulting mixture was then filtered using UNI-Sci qualitative filter paper No. 102 (Cat. No. US-C25102090PH). A 15 mL aliquot of the filtrate was used immediately for antibacterial MIC testing against SA, PA, and SE. The remaining filtrate was dried in an oven at 80 °C overnight to obtain the solid M8/ZnO composite for morphological (SEM), structural (XRD), and chemical (FTIR) characterization.

### 2.5. Evaluating Antibacterial Activities of Polymer Blends

The *Staphylococcus aureus* strain ATCC 29523 and *Salmonella enterica* ATCC 14028 were provided by the School of Biotechnology, International University—Vietnam National University (Ho Chi Minh City, Vietnam). The *Pseudomonas aeruginosa* strain ATCC 9027 was obtained from the Research Center for Infectious Disease, International University—Vietnam National University (Ho Chi Minh City, Vietnam). These bacteria were grown in Mueller Hinton Broth (Himedia Laboratories, Maharashtra, India) for 24 h at 37 °C under an aerobic environment. Microscopic observation, as well as Gram-staining, was performed to check for any contaminants.

A minimum inhibitory concentration (MIC) assay was performed, which is in accordance with Linh et al.’s study, with the change in wavelength from 600 nm to 595 nm. The instrument used was a BioTek ELx808 (BioTek Instruments, Winooski, VT, USA) [38].

## 3. Results and Discussion

### 3.1. Characterization of M8 and M8/ZnO

To analyze the morphology, including the particle size, of ZnO, SEM analysis was conducted. Additionally, to confirm the existence of the ZnO particles, XRD was used. To determine the bonding between M8 and ZnO in the composite of M8/ZnO, FTIR was used.

#### 3.1.1. Scanning Electron Microscopes (SEMs)

**Figure 1:**
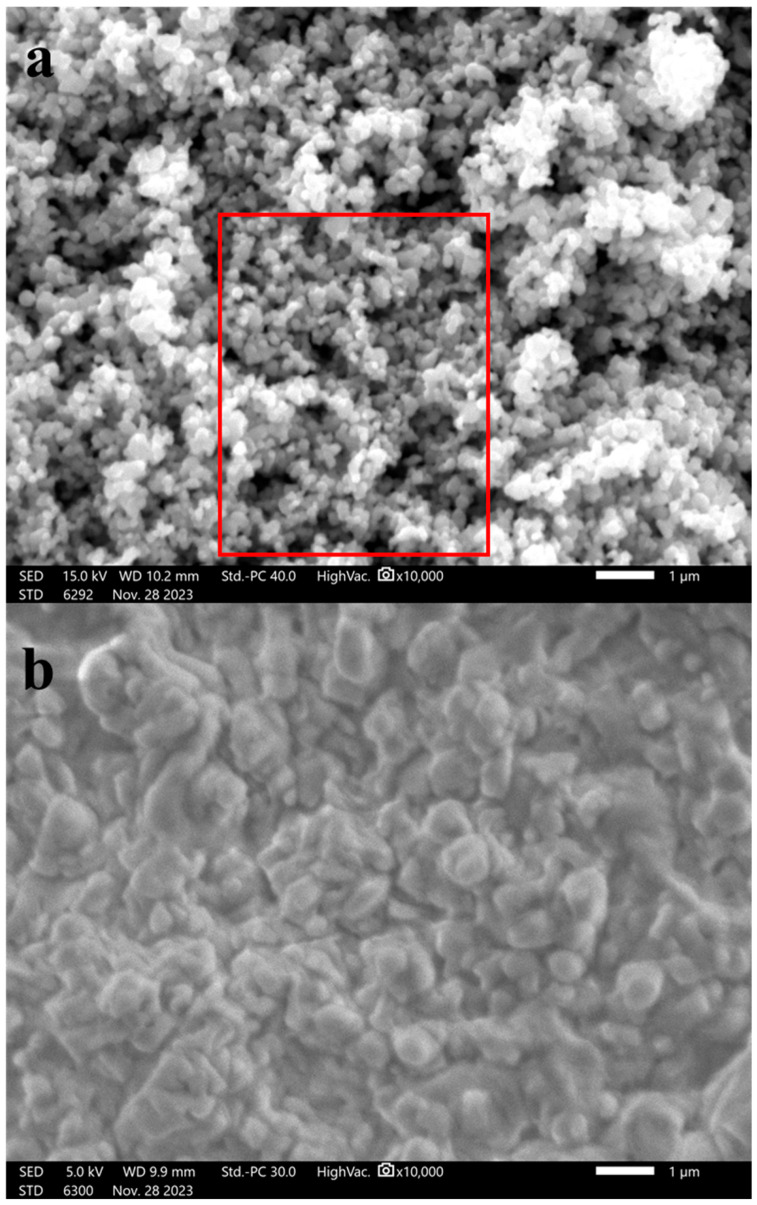
SEM images of (**a**) ZnOPs and (**b**) M8/ZnO.

A scanning electron microscope (SEM) (JEOL JSM-IT200, Tokyo, Japan) was used to characterize ZnOPs and M8/ZnO, as Figure 1 demonstrates.

As shown in Figure 1a, the ZnOPs were shown to be spherical and aggregated. By using ImageJ software (1.54G), a section of the SEM image (red box) was duplicated, and the image threshold was modified, as shown in Figure S1. Then, the size of the particles was determined to be 181.8 nm using the ImageJ function ‘Analyze Particles’, as shown in Figure S2. As shown in Figure 1b, the ZnOPs were not shown clearly anymore. This phenomenon is caused by the coverage or coating of the polymer blend, M8, on the outside of the ZnO particles. In other words, the polymer blend (M8) spread over and encapsulated the ZnO particles, reducing the visibility of their original morphology. However, the confirmation of the structure of M8/ZnO must be determined using FTIR, and the types of ZnO particles must be confirmed using XRD.

#### 3.1.2. FTIR

**Figure 2:**
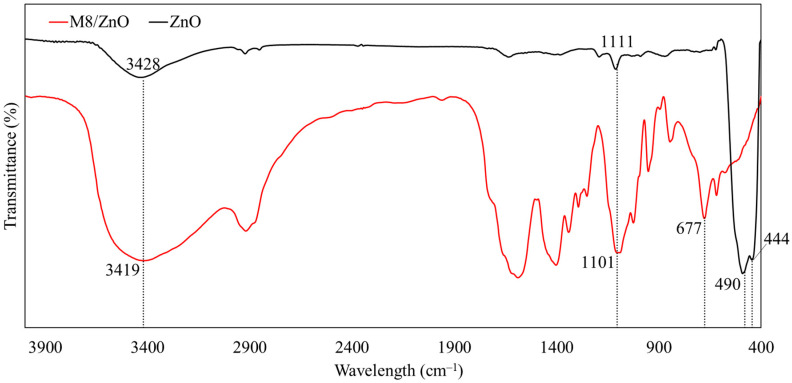
FTIR spectra of ZnO and M8/ZnO.

The functional groups and bonding in the ZnO and M8/ZnO were determined using a Fourier transform infrared (FTIR) spectrometer (Tensor 27, Bruker, Billerica, MA, USA) in the spectral range from 400 to 4000 cm^−1^. The result of the FTIR analysis is given in Figure 2 and Table 1.

As shown in Table 1 and Figure 2, the shift in absorption bands at 1525 cm^−1^ and 1430 cm^−1^ indicates the absorption of PVA into the ZnO NPs [39]. The band at 1341 cm^−1^ corresponds to the PVP–ZnO covalent bonds, and the lack of bands around 400 cm^−1^ is caused by the low concentration of ZnO in the polymer matrix [40]. The band at 1100 cm^−1^ might be the C–O stretching vibrations, which are caused by the intermolecular interactions between PVA and PVP [41]. A band around 600 cm^−1^ indicates the presence of ZnOPs and the stretching of ZnO in PVA [42,43]. The band of 1024 cm^−1^ corresponds to C–OH stretching, which implies that the level of PVA decreased in the composite because the ZnOPs induced imperfections.

The reduction in Zn–O band intensity around ~677 cm^−1^ indicates the partial coverage of ZnO by the polymer network, consistent with the SEM findings. Additionally, the band at ~2900 cm^−1^ does not belong to ZnO. It belongs to the polymer blend, M8. Specifically, it is the asymmetric C–H stretching vibration [47,48]. These spectral changes demonstrate that the M8 components (PVA, PVP, PEG, and chitosan) participate in stabilizing or coordinating the ZnO surface, forming the intended composite. Additionally, the FTIR analysis shows shifts in the C–O, O–H, and C=O bands, indicating hydrogen bonding and coordination interactions (particularly between PVP and Zn^2+^), but there is no evidence of covalent bonding. Thus, M8 and ZnO are connected primarily through surface interactions rather than chemical covalent bonds.

#### 3.1.3. Energy-Dispersive X-Ray Spectroscopy (EDS)

The elemental components of M8/ZnO and ZnO were examined via energy dispersive spectroscopy (EDS) analysis using JEOL JED-2300, Tokyo, Japan. The results are shown in Figure S3, while Table 2 and Table 3 illustrate the M8/ZnO and ZnO EDS analysis.

As shown in Table 2 and Table 3, the ZnO content reported in Table 3 was obtained directly from the EDS instrument’s integrated quantification software, which automatically converts elemental mass percentages into oxide compositions. No manual calculation was performed.

As shown in Table 2 and Table 3, the purity of ZnOPs was 99.03 ± 1.71% and the M8/ZnO has 7.45 ± 0.93% ZnO in the polymer blend. The data confirm that the ZnOPs are largely ZnO, reflecting the high purity of the synthesized particles. This indicates that the rest of the matrix is comprised of polymers (PEG, PVP, PVA, and CS). However, a very small amount of trace elements (Al_2_O_3_, SiO_2_, and SO_3_) was detected in the ZnOPs samples. This might be from the chemical manufacturers or from the process of synthesis (i.e., drying process using glassware). Importantly, the detectable Zn signal confirms that ZnO is present and successfully incorporated into the M8 matrix.

#### 3.1.4. X-Ray Diffraction (XRD) Analysis

**Figure 3:**
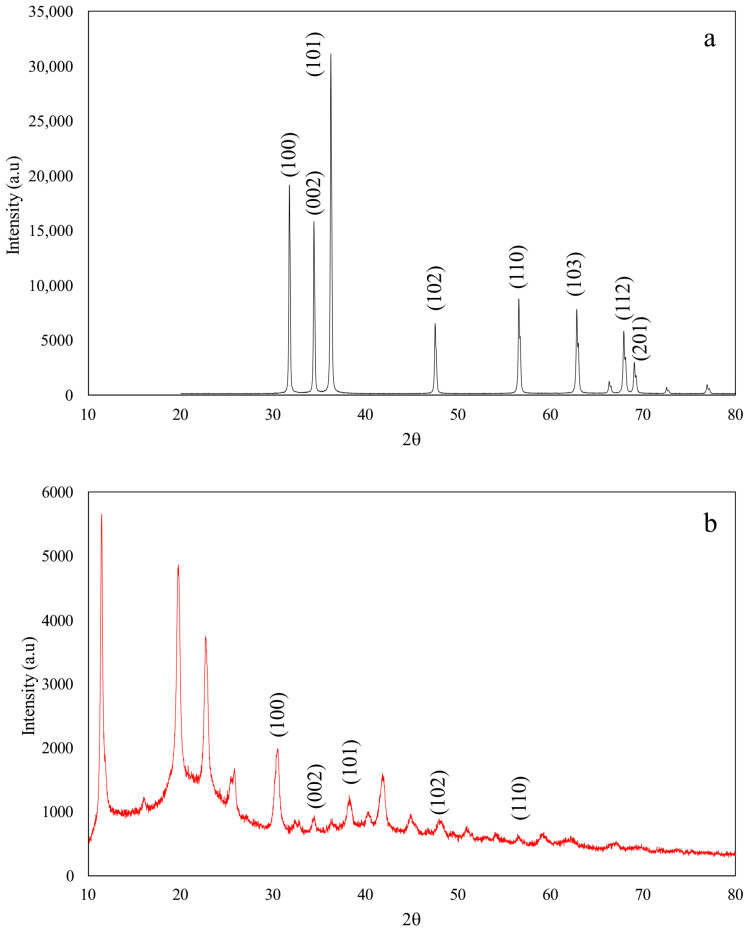
XRD spectra of (**a**) ZnOPs and (**b**) M8/ZnO.

Based on the X-ray diffractograms of ZnOPs, as seen in Figure 3a, it was determined that the nanoparticles were ZnOPs since they showed peaks at 2θ positions of 31.75°, 34.41°, 36.24°, 47.52°, 56.57°, 62.84°, 67.93°, and 69.06°. This indicates that the ZnO has the wurtzite structure—the most common structure—which is stable at room temperature and atmospheric pressure [49]. These angles were identified (100), (002), (101), (102), (110), (103), (112), and (201) planes, respectively, which are similar to those found in other publications [45,50,51]. Based on Figure 3b, the 2θ peaks of 34.37°, 36.23°, 56.53°, 62.24°, and 67.07° matched with the peaks of ZnOPs in Figure 3a. This indicates that the M8 successfully covered the ZnOPs. On the other hand, the peaks at 31.75° and 69.06° disappeared. The reason for this was unknown. However, the peak intensity of ZnO decreased, indicating that the ZnOPs were covered by polymers. This might explain the disappearance of the peaks. This phenomenon is widely reported in polymer–nanoparticle systems and supports successful surface modification.

### 3.2. Antimicrobial Activities

**Figure 4:**
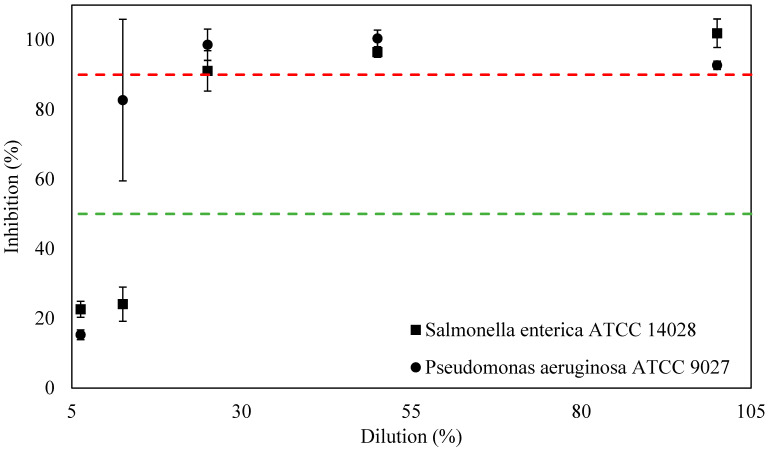
Inhibition percentage of M8 against SE, SA, and PA. The green and red line indicates MIC50 and MIC90, respectively.

To determine the antimicrobial activity of M8 and M8/ZnO against SA, PA, and SE, the minimum inhibition concentration method was employed. The MIC results can be seen in Figure 4.

As shown in Figure 4, the MIC50, which is the minimum inhibition concentration required to have at least 50% inhibition percentage, is 25% and 12.5% of M8 when in direct contact with SE and PA, respectively. However, in the case of M8 with PA at 12.5%, the standard deviation was quite large, indicating that the more reliable data is found at 25%. Hence, the MIC50 of M8 against SE and PA is 25%.

In the case of having at least 90% inhibition percentage, MIC90 is at 25% and 25% when M8 was in direct contact with SE and PA, which is similar to MIC50. According to Linh et al., M8 has larger MIC values against SE and PA than SA [38]. On the other hand, the MIC90 of M8 against SE, PA, and SA is the same [38].

After determining the MIC50 and MIC90 values of M8 against SE and PA, M8 was modified with ZnO nanoparticles. The MIC results are shown in Figure 4.

**Figure 5:**
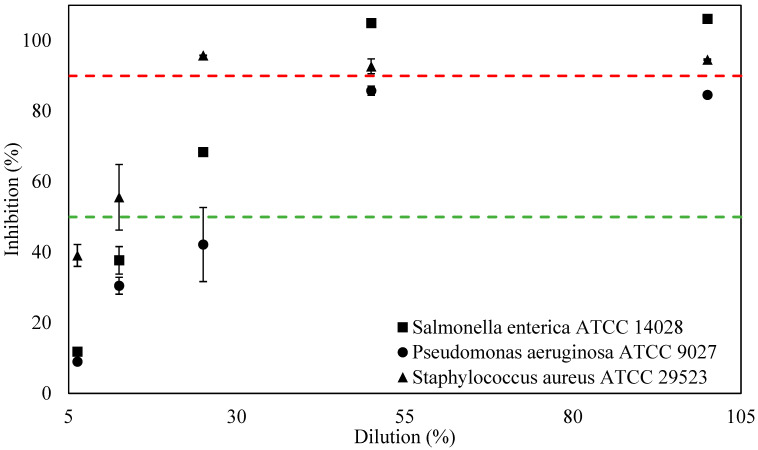
Inhibition percentage of M8/ZnO against SE, SA, and PA. The green and red line indicates MIC50 and MIC90, respectively. In short, the MIC50 and MIC90 of M8 and M8/ZnO are shown in Table 4.

Based on Figure 5, the MIC50, which is the minimum inhibition concentration to have at least 50% inhibition percentage, is at 25%, 12.5%, and 50% of M8/ZnO when in direct contact with SE, SA, and PA, respectively. According to Linh et al., M8/ZnO has more similar MIC50 values against SA than M8 [38].

In the case of having at least 90% inhibition percentage, the MIC90 of M8/ZnO against SE and SA is 100% and 25%, respectively. However, M8/ZnO does not have MIC90 in the case of PA. Comparing the MIC90 of M8/ZnO against SA with M8, M8/ZnO has the same MIC90 value [38].

As shown in Table 4, the MIC50 values indicate that M8 and M8/ZnO have antimicrobial ability against both Gram-positive and -negative bacteria (i.e., SE, SA, and PA). However, in the presence of ZnO in M8, the composite increases the MIC50 values when the composite is in direct contact with PA. In the presence of ZnO in M8, the MIC90 values also increase in the case of SE. This indicates that M8/ZnO might not be as effective against Gram-negative bacteria as M8, especially against PA. The lack of MIC90 values of M8/ZnO against PA is similar to results in the literature [52,53,54]. Based on the literature, it seems possible that antibacterial mechanisms can be caused by the interaction of bacteria and the polymers, as well as ZnO particles. The novel composite consists of M8, which is the blend of PEG, PVP, PVA, and CS. Based on the blend, PEG and PVP minimize the adhesion of bacteria to the surface [55]. Combining it with CS, which has multiple amino groups (positively charged), destroys the negatively charged bacterial cell wall by disrupting the RNA synthesis process [56]. Additionally, ZnO might also contribute to antibacterial activity. In the case of only the presence of ZnO particles, three possible antibacterial mechanisms are the increase in reactive oxygen species (ROS) in bacteria; the interaction of particles with bacteria, which disrupts the bacterial cell wall; and the release of Zn^2+^ ions [57,58,59]. Specifically, in the case of SA, ZnO increased the ROS, which was later deposited on the surface or accumulated in the SA cells’ cytoplasm [60]. As shown in Table 4, M8/ZnO exhibits superior antibacterial properties against Gram-positive bacteria compared to Gram-negative bacteria, a finding that is similar to that of other research [19,61]. Gram-negative bacteria have bilayer cell walls, which consist of an outer layer of lipopolysaccharide [56]. This outer layer limits the permeability of the particles, which can act as a shield against ZnO particles [56,62]. However, these explanations are just based on many studies and the known antibacterial mechanism of ZnO particles; it is also worth noting that novel materials have not been found [62].

Moreover, in most cases, in vitro results do not correspond to in vivo activities. The findings of this investigation imply that M8 and M8/ZnO may have inhibitory properties against SE, PA, and SA. As a result, more in vivo investigations are required before using them in the healthcare field.

Interestingly, ZnO did not enhance the antibacterial performance of M8, and in some cases, particularly for P. aeruginosa, the inhibition was substantially reduced. This observation implies that the introduction of ZnO alters the functional environment of the M8 polymer blend. Because M8 relies on the combined effects of PVA, PVP, PEG, and chitosan, the addition of inorganic particles may change the exposure or mobility of these components, limit the availability of active sites, or influence how the mixture interacts with bacterial membranes. Although determining the precise mechanism is beyond the scope of this work, the results demonstrate that ZnO does not universally improve polymer-based antibacterial systems. Instead, ZnO can attenuate activity depending on the bacterial type and dilution conditions, highlighting the importance of understanding compatibility and formulation effects in polymer–nanoparticle composites.

## 4. Conclusions

Based on previous publications, a polymer blend (M8) was modified using zinc oxide particles. ZnO particles have an average size of 181.8 nm. The novel composite was used for its antimicrobial activity against *Staphylococcus aureus*, *Pseudomonas aeruginosa*, and *Salmonella enterica*. Based on the minimum inhibitory concentration experiments, the MIC50 and the MIC90 values showed that M8 has antimicrobial activity against both Gram-positive and Gram-negative bacteria (*Staphylococcus aureus*, *Pseudomonas aeruginosa*, and *Salmonella enterica*). The MIC50 values against PA and SE are 12.5 and 12.5% of M8 when in direct contact with the bacteria, respectively. The MIC90 values against PA and SE are both at 25% of M8 when in direct contact with the bacteria. Compared to M8, M8/ZnO has a different MIC50 against PA, which is 50% of M8/ZnO. For SE, the MIC90 of M8/ZnO is 50% of M8/ZnO. For PA, M8/ZnO does not have MIC90 values. Additionally, the incorporation of ZnO did not improve the antibacterial activity of M8 and, in some cases, resulted in reduced inhibition. Overall, the results show that the surface modification of ZnO with the M8 blend alters both its physicochemical and biological properties, demonstrating a promising yet selective antibacterial potential. This provides a foundation for the future optimization of particle ratios, polymer compositions, and targeted antibacterial applications.

## Figures and Tables

**Table 1:** List of FTIR absorption bands of ZnO and M8/ZnO.

Chemicals	Wavelength (cm^−1^)	Functional Group	References
M8/ZnO	3419	–OH stretching vibration of PVA, PVP, PEG secondary –NH groups of CS + AA	[38]
	1589–1402	Absorption of PVA on ZnONPs	[39]
	1341	PVP–ZnO covalent bonds	[40]
	1293	C–H bond in pyranose ring of CS + AA	[38]
	1101	C–O stretching vibration	[41]
	1024	C–OH stretching vibration	[41]
	677–678	High-purity ZnONPs Stretching vibration of Zn–O in PVA	[42,43]
ZnO	3428	Stretching vibration of O–H groups	[44]
	1111	C–O stretching band	[44]
	444–490	Stretching vibration of Zn–O bond	[45,46]

**Table 2:** Elemental composition mass (%).

Element	ZnO	M8/ZnO
C	-	44.90 ± 0.27
N	-	2.83 ± 0.26
O	18.93 ± 0.22	49.16 ± 0.57
Al	0.16 ± 0.03	0.18 ± 0.03
Si	0.09 ± 0.03	-
S	0.22 ± 0.03	-
Zn	80.59 ± 1.41	2.93 ± 0.37
Total	100	100

**Table 3:** Chemical formula composition mass (%).

Chemical Formula	ZnO	M8/ZnO
C	-	79.86 ± 0.48
N	-	12.06 ± 1.12
O	-	
Al_2_O_3_	0.27 ± 0.06	0.63 ± 0.10
SiO_2_	0.25 ± 0.06	-
SO_3_	0.45 ± 0.07	-
ZnO	99.03 ± 1.71	7.45 ± 0.93
Total	100	100

**Table 4:** MIC results of M8 and M8/ZnO against SE, SA, and PA [38].

Bacteria	MIC50		MIC90	
	M8	M8/ZnO	M8	M8/ZnO
SE (Gram-negative)	25%	25%	25%	50%
SA (Gram-positive)	12.5% [38]	12.5%	12.5% [38]	25%
PA (Gram-negative)	12.5%	50%	25%	-

## Data Availability

All data that support the findings of this study are included within the article.

## Supplementary Materials

The following supporting information can be downloaded at https://www.mdpi.com/article/10.3390/polym17243283/s1: Figure S1: ZnO particles SEM image processed using ImageJ (1.54G). Figure S2: ZnO particles SEM image processed using ImageJ (1.54G). Figure S3: EDS spectra of (a) ZnOPs and (b) M8/ZnO.
