# Supplementary material for: Surface Modifications of Zinc Oxide Particles with Chitosan, Polyethylene Glycol, Polyvinyl Alcohol, and Polyvinylpyrrolidone as Antibacterial Agents
Source: Polymers (Basel). 2025 Dec 11;17(24):3283. doi: 10.3390/polym17243283 (PMC12737094; doi:10.3390/polym17243283)
Supplement: Supplementary file 1 [file polymers-17-03283-s001.zip › polymers-4022713-supplementary.pdf]

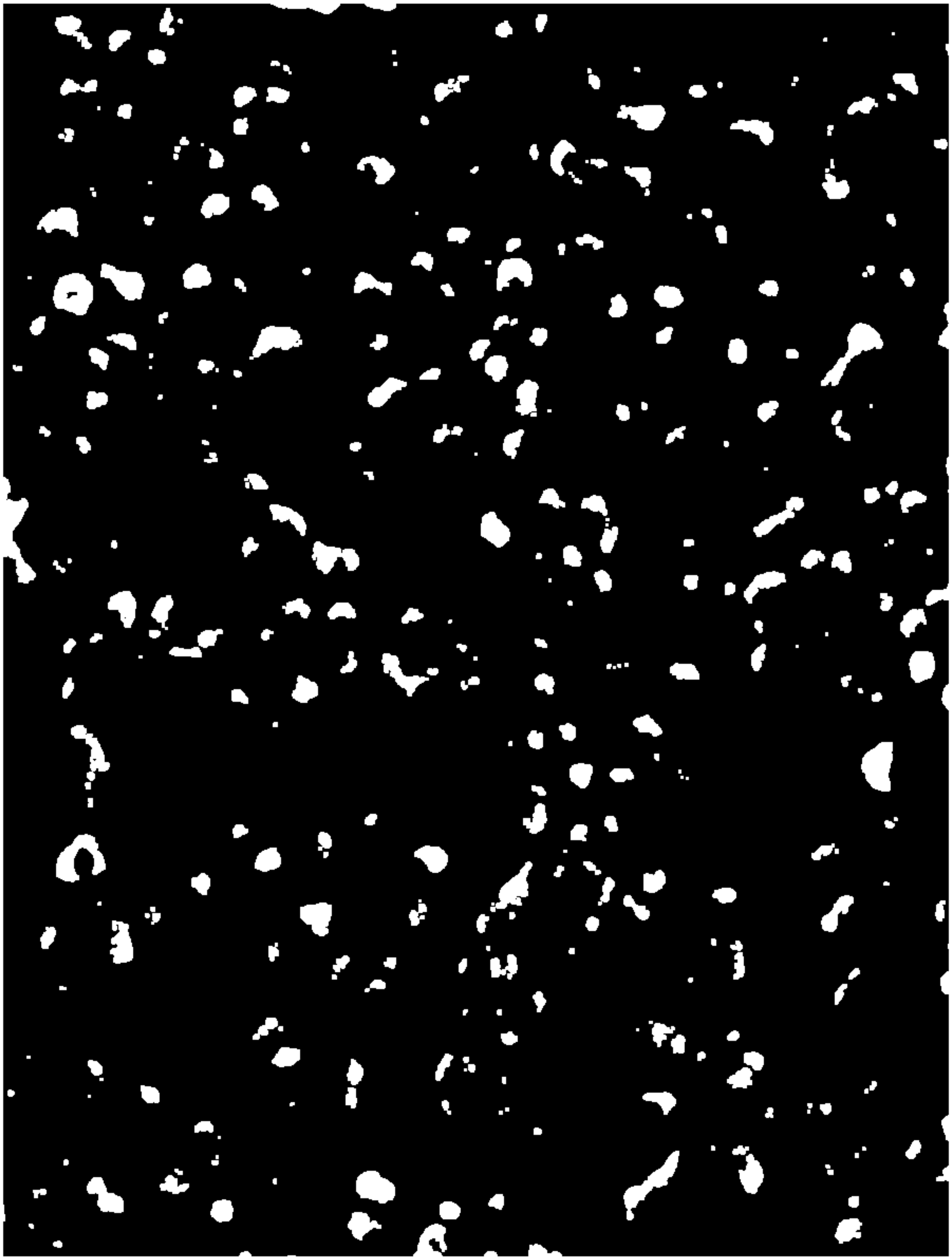

Figure S1. ZnO particles SEM image processed using ImageJ (adjust Threshold)

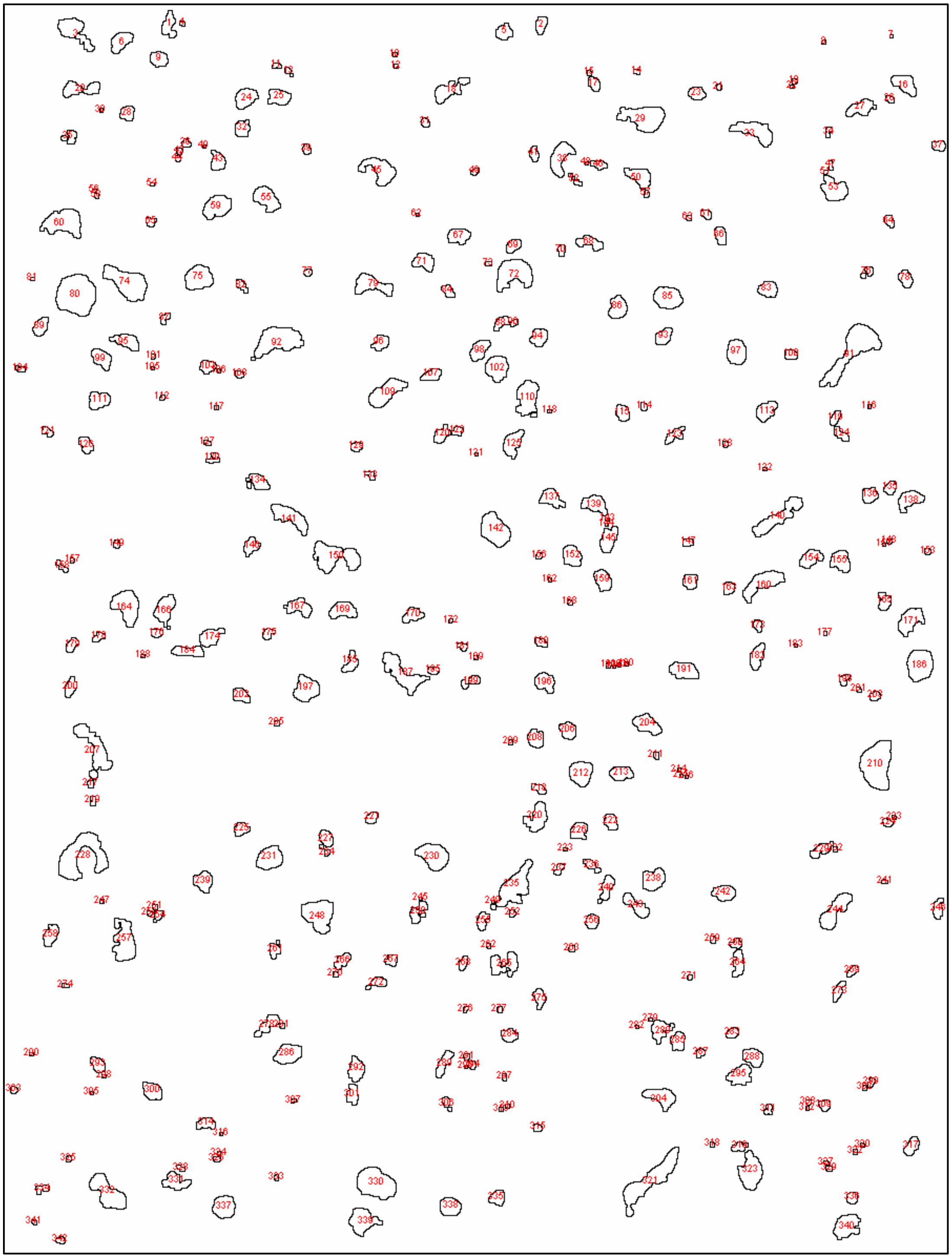

Figure S2. ZnO particles SEM image processed using ImageJ (Analyze particles)

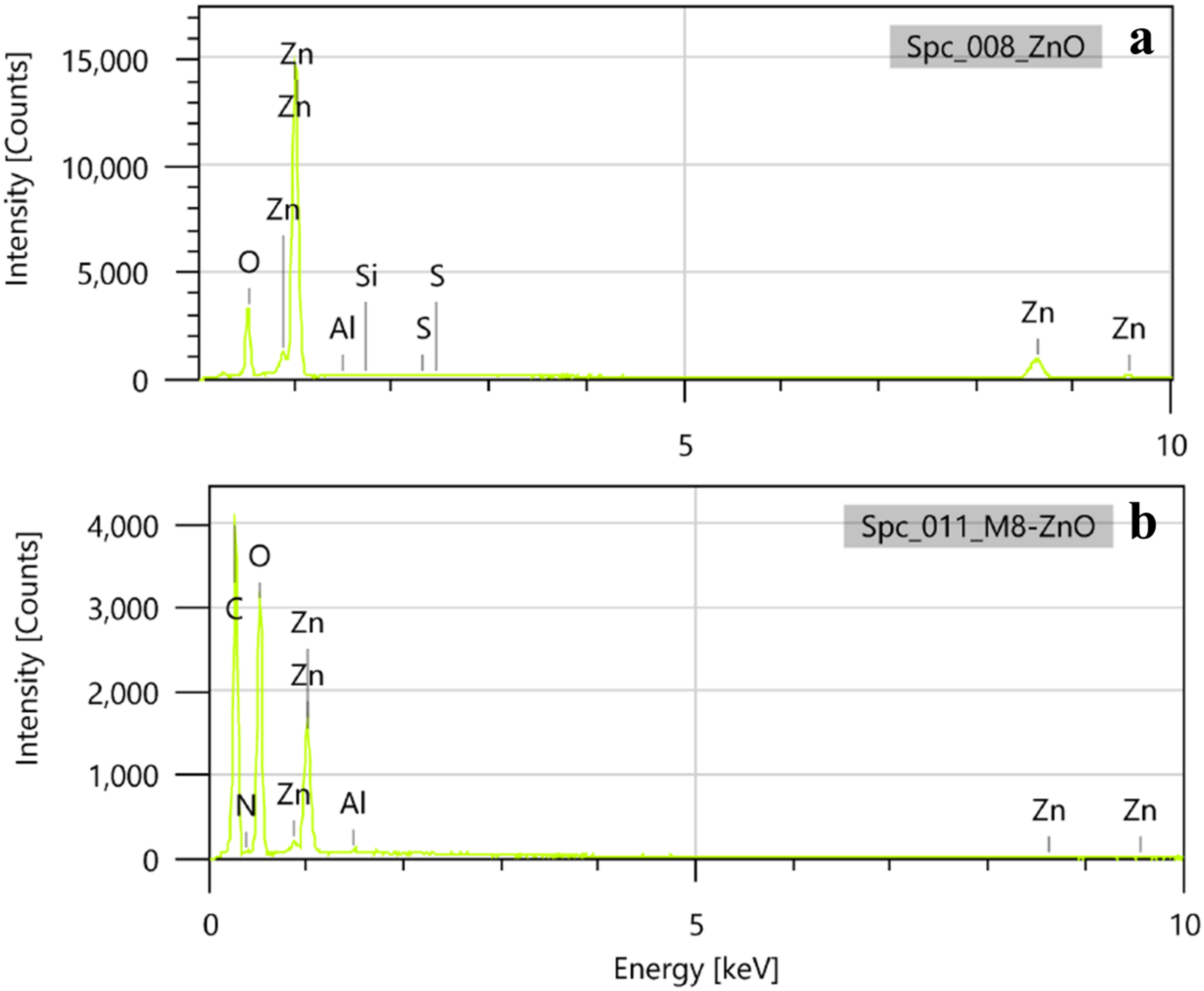

Figure S3. EDS spectra of (a) ZnOPs and (b) M8/ZnO
